# Supplementary material for: ApoE Isoforms Inhibit Amyloid Aggregation of Proinflammatory Protein S100A9
Source: Int J Mol Sci. 2024 Feb 9;25(4):2114. doi: 10.3390/ijms25042114 (PMC10889306; doi:10.3390/ijms25042114)
Supplement: Supplementary file 1 [file ijms-25-02114-s001.zip › ijms-2813501-supplementary.pdf]

ApoE Isoforms Inhibit Amyloid Aggregation of Proinflammatory Protein S100A9

Supplementary Information

Shamasree Ghosh [1], Shanmugam Tamilselvi [1], Chloe Williams [2], Sanduni W. Jayaweera [3], Igor A. Iashchishyn [1], Darius Šulskis [4], Jonathan D. Gilthorpe [2], Anders Olofsson [3], Vytautas Smirnovas [4], Željko M. Svedružić [5,†] and Ludmilla A. Morozova-Roche [1,*]

[1] Department of Medical Biochemistry and Biophysics, Umeå University, SE-90187 Umeå, Sweden;
shamasree.ghosh@umu.se (S.G.); tamilselvi.shanmugam@umu.se (S.T.); igor.iashchishyn@umu.se (I.A.I.)

[2] Department of Medical and Translational Biology, Umeå University, SE-90187 Umeå, Sweden;
chloe.williams@umu.se (C.W.); jonathan.gilthorpe@umu.se (J.D.G.)

[3] Department of Clinical Microbiology, Umeå University, SE-90187 Umeå, Sweden;
sanduni.jayakodi@umu.se (S.W.J.); anders.olofsson@umu.se (A.O.)

[4] Institute of Biotechnology, Life Sciences Center, Vilnius University, LT-10257 Vilnius, Lithuania;
darius.sulskis@gmc.vu.lt (D.Š.); vytautas.smirnovas@bti.vu.lt (V.S.)

[5] Department of Biotechnology, University of Rijeka, HR-51000 Rijeka, Croatia

[*] Correspondence: ludmilla.morozova-roche@umu.se

[†] Professor Željko M. Svedružić passed away on 12 April 2023 and this article is dedicated to his memory.

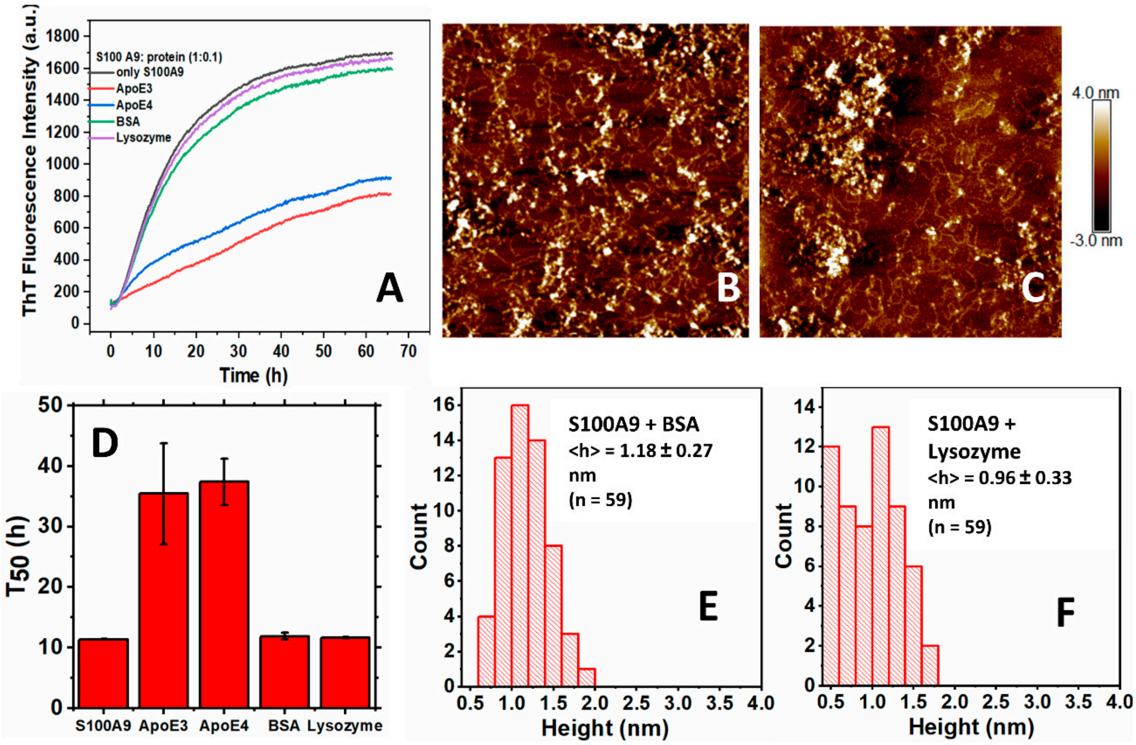

Supplementary Figure S1. Non-specific effect of bovine serum albumin and lysozyme on the amyloid aggregation of S100A9 compared to ApoE3 and ApoE4. (A) Aggregation kinetics of S100A9 in the absence of any additives (black line) and in the presence of ApoE3 (red line), ApoE4 (blue line), bovine serum albumin (green line) and lysozyme (purple line) monitored by ThT fluorescence assay. 50 μM S100A9 and 5 μM of each protein were incubated in PBS buffer, containing 1 mM sodium azide and 20 μM ThT, at 42 °C, non-shaking condition. ThT fluorescence was excited at 430 nm and its emission fluorescence was recorded at 485 nm. (B,C) AFM images of S100A9 amyloids formed in the presence of bovine serum albumin and lysozyme, respectively, at the end of 70 h aggregation kinetics as shown in (A). The scan sizes are 2 × 2 μm. *z*-scale bar is shown on the right from the images. (D) Half-time (T_{50}) corresponds to time, when ThT fluorescence intensity reaches half of plateau value. The samples are indicated below *x*-axis. (E,F) The distributions of height of S100A9 fibril incubated in the presence of bovine serum albumin and lysozyme, respectively, measured in AFM cross-sections. The mean values of fibril heights of ~ 1 nm are shown in the figure insets.

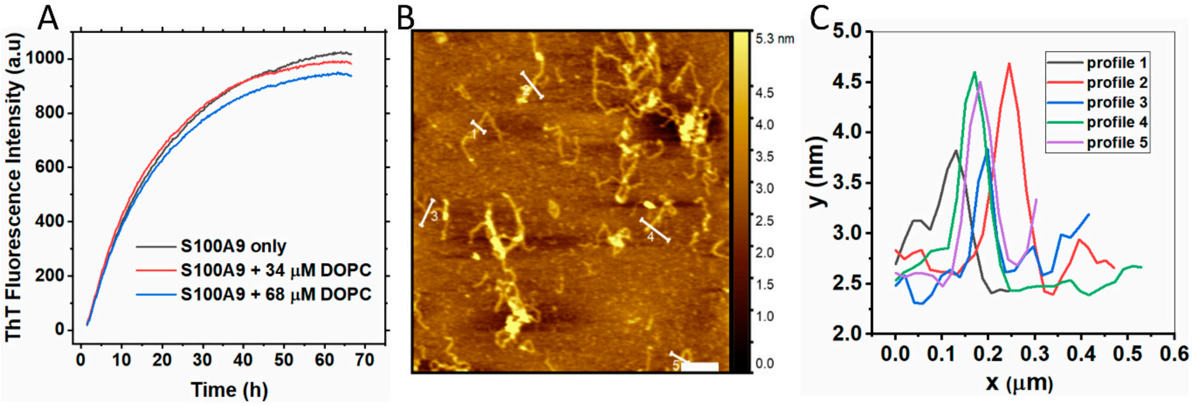

Supplementary Figure S2. Aggregation of S100A9 in presence of DOPC lipid particles monitored by ThT fluorescence assay and AFM imaging. (A) Aggregation kinetics of 50 μM S100A9 in the absence (black line) and presence of DOPC lipid particles (red line − 34 μM and blue line −68 μM) in PBS, 1 mM NaN_3, 42 °C monitored by ThT fluorescence. 20 μM ThT was used. (B) AFM image of S100A9 aggregates after 70 h incubation in the presence of 68 μM DOPC particles. The scan size is 5 × 5 μm. The *z*-scale in AFM image is indicated by bar with a color gradient from black to light yellow. (C) Representative AFM cross sections of S100A9 fibrils measured at the end of aggregation. The fibrils are straight and their height varies from 1 to 2 nm.

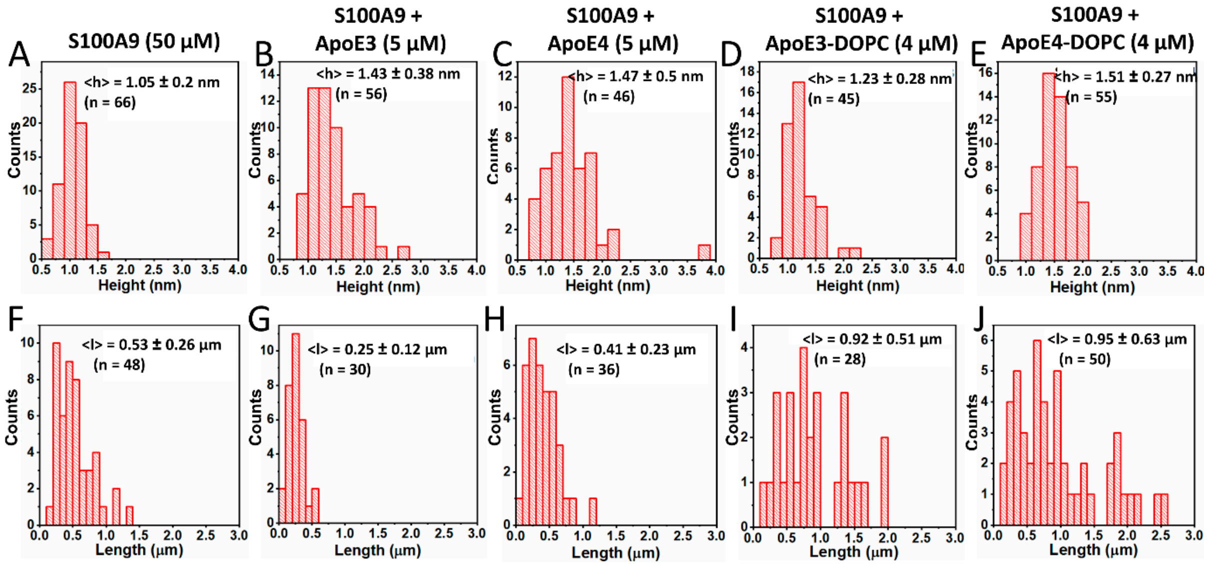

Supplementary Figure S3. Parameters of S100A9 fibrils measured in AFM cross-sections after 70 h aggregation. Distributions of height of S100A9 fibrils measured in AFM cross-sections after 70 h aggregation: (A) S100A9 alone; (B) S100A9 + ApoE3; (C) S100A9 + ApoE4; (D) S100A9 + ApoE3-DOPC and (E) S100A9 + ApoE4-DOPC. Mean heights (h) and standard deviations are shown in the corresponding insets. Distributions of length of S100A9 fibrils after 70 h aggregation: (F) S100A9 alone; (G) S100A9 + ApoE3; (H) S100A9 + ApoE4; (I) S100A9 + ApoE3-DOPC and (J) S100A9 + ApoE4-DOPC. Mean lengths (l) and standard deviations are shown in the corresponding insets.

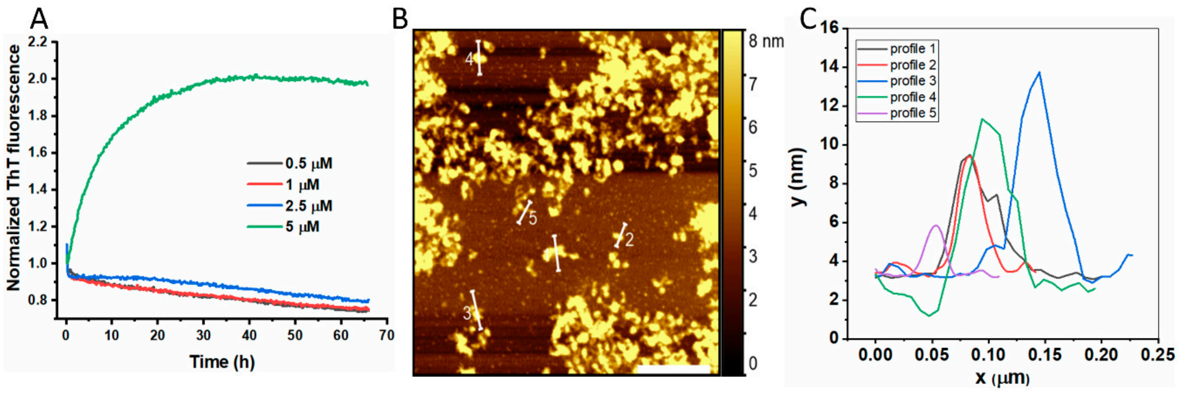

Supplementary Figure S4. Aggregation of ApoE3 alone monitored by ThT fluorescence assay and AFM imaging. (A) Aggregation kinetics of increasing concentrations of ApoE3, viz. 0.5 μM (black line), 1 μM (red line), 2.5 μM (blue line) and 5 μM (green line) in PBS, 1 mM NaN_3, at 42 °C monitored by ThT fluorescence. 20 μM ThT was used. (B) AFM image of 5 μM ApoE3 aggregates after 70 h incubation at the above conditions. The scan size is 2 × 2 μm. The *z*-scale of AFM image is indicated by a bar with a color gradient from black to light yellow. (C) Representative cross sections of ApoE3 aggregates at the end of aggregation. The aggregates are heterogeneous with heights varying from 2 to 8 nm. They are not fibrillary in morphology.

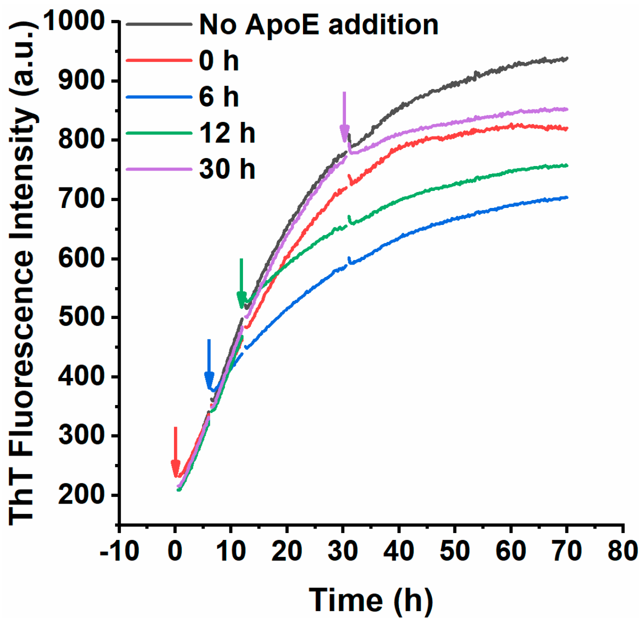

Supplementary Figure S5. Inhibiting effect of ApoE3-DOPC on the amyloid formation of S100A9 upon addition at different time points during aggregation. Aggregation kinetics of 50 µM S100A9 (black line), with the addition of 3 µM ApoE3-DOPC at t = 0 (red line), at t = 6 h (blue line), at t = 13 h (green line) and at t = 30 h (purple line). Arrows indicate the time points, when ApoE3-DOPC was added. 20 µM ThT was used to monitor the aggregation kinetics. The aggregation was performed at 42 °C.

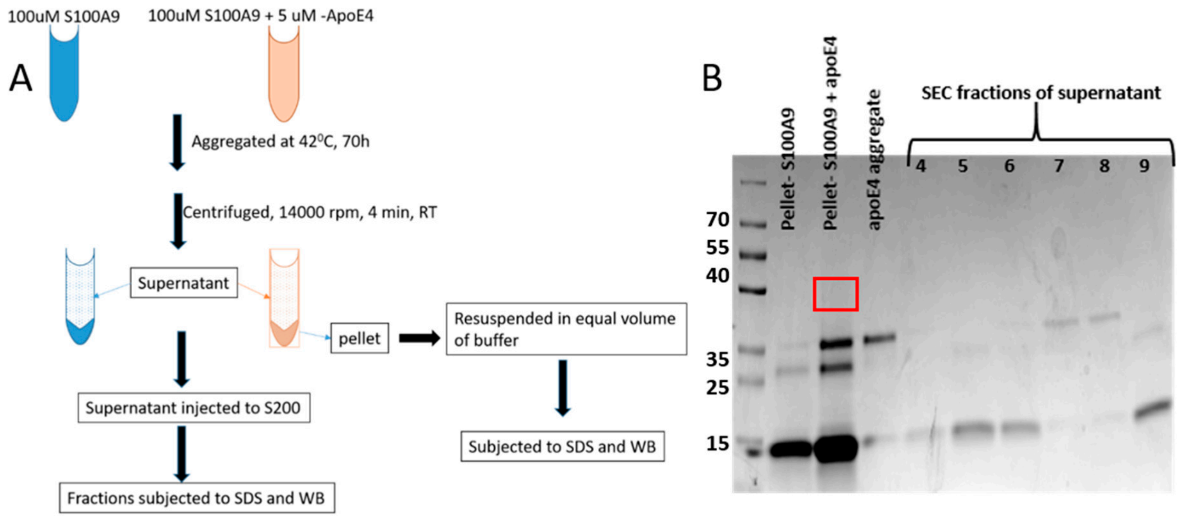

Supplementary Figure S6. Schematic presentation of the procedure used for S100A9-ApoE complex formation and purification. (A) Schematic presentation of the protocol for S100A9-ApoE4 complex formation and purification. The details of the experiment are presented in “Materials and Methods”. (B) SDS-PAGE of the pellets collected prior to the elution and the supernatant collected during the elution through size exclusion chromatography upon S100A9, ApoE4 and S100A9 and ApoE4 aggregation. The chromatogram is shown in Figure 4. The band corresponding to ~15 kDa in all relevant fractions is S100A9 monomer, the band of ~25 kDa corresponds to S100A9 dimer, and the band of ~35 kDa corresponds to ApoE4. The band corresponding to the complex of S100A9-ApoE4 of ~ 50 kDa in the pellet as marked with red square is very faint, when stained with Coomassie blue. 100 μM S100A9, 5 μM ApoE4 and their mixture were incubated in PBS, 1 mM NaN_3 at 42 °C for 70 h. Protein samples were subjected to 4-20% gradient SDS-PAGE under reducing conditions and stained with Coomassie blue.

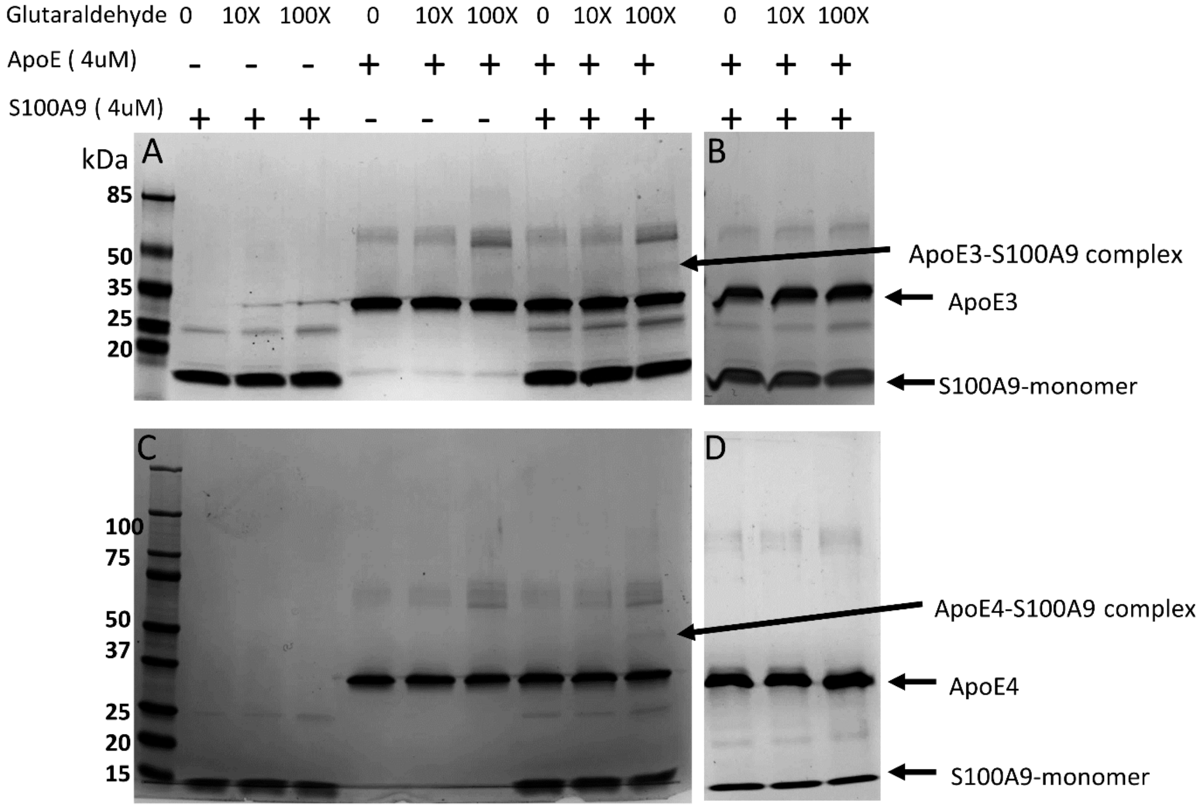

Supplementary Figure S7. Complex formation between native S100A9 and ApoE isoforms in the presence of increasing concentrations of glutaraldehyde cross-linker. SDS PAGE of (A) 4 μM S100A9 in the absence and presence of 4 μM ApoE3 and increasing concentrations of glutaraldehyde from 0 to 100X; (B) S100A9 mixed with ApoE3 lipidated with DOPC and increasing concentrations of glutaraldehyde; (C) 4 μM S100A9 in the absence and presence of 4 μM ApoE4 and increasing concentrations of glutaraldehyde from 0 to 100X; (D) S100A9 with ApoE4 lipidated with DOPC and increasing concentrations of glutaraldehyde. The samples were subjected to 4-20% gradient SDS-PAGE under reducing conditions and detected by Coomassie blue staining.

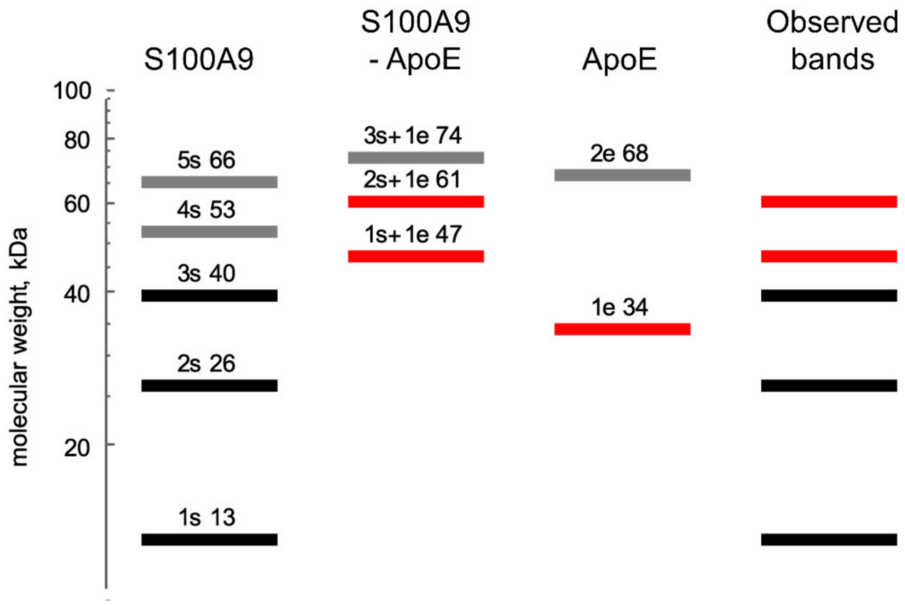

Supplementary Figure S8. Schematic presentation of theoretically predicted and experimentally observed matching patterns of molecular masses upon S100A9-ApoE complex formation. The first three columns show experimentally observed in SDS-PAGE and theoretically predicted bands of the corresponding complexes. The black bands correspond to molecular masses of the experimentally observed homo-complexes of S100A9, red bands – to experimentally observed ApoE alone and in the complexes with S100A9 and grey bands – to theoretically calculated masses of higher order complexes. The fourth column corresponds to the Western blot bands observed under the cross-linking conditions by using anti-S100A9 antibodies, which demonstrate the full coincidence between the theoretically calculated and experimentally observed bands.

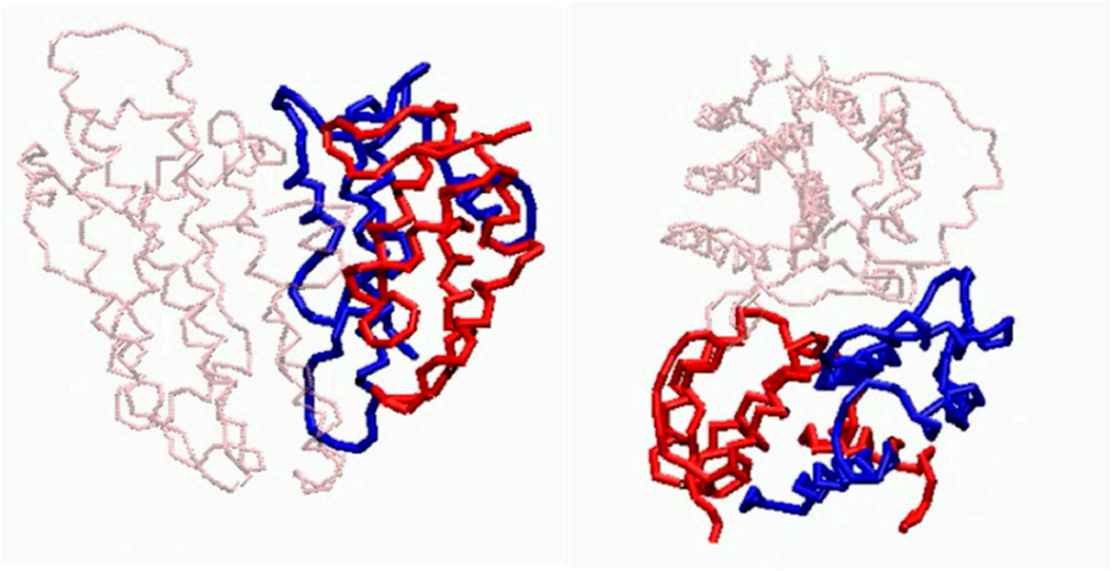

Supplementary Figure S9. Coarse grained simulation of ApoE4 and S100A9 dimer complex formation after 20 µs simulation shown in two projections. The backbone of ApoE4 is presented in pink and the monomers of S100A9 dimer are shown in red and blue, respectively.

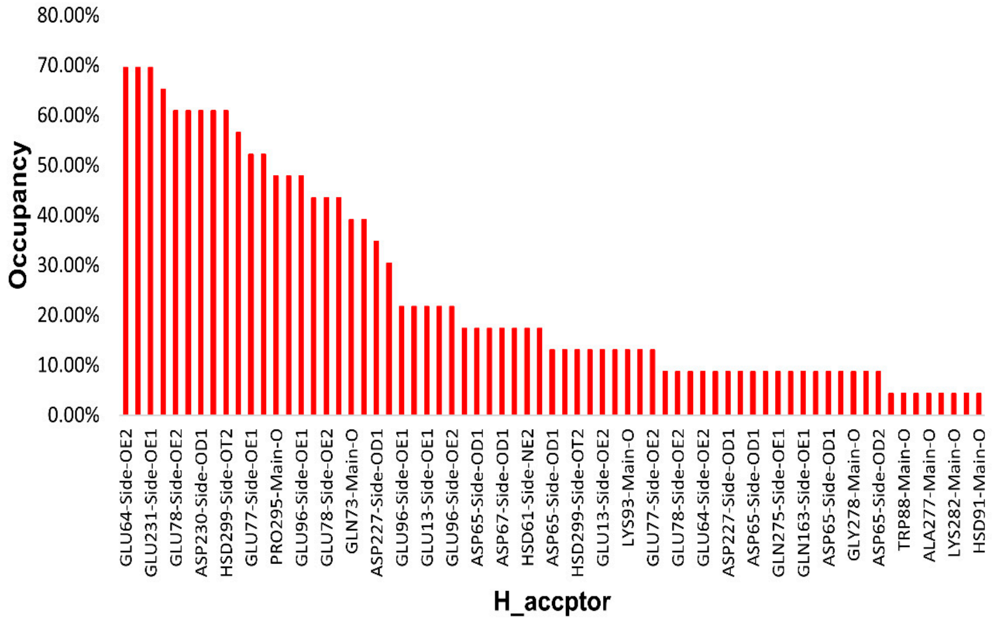

Supplementary Figure S10. Occupancy of donor-acceptor pairs in S100A9-ApoE4 complex after 20 µs of coarse-grained simulation. Percentage of occupancy is shown along *y*-axis and the acceptor in the donor-acceptor pairs with such occupancy – along *x*-axis. The complete list of donor-acceptor pairs is presented in Supplementary Table 1.

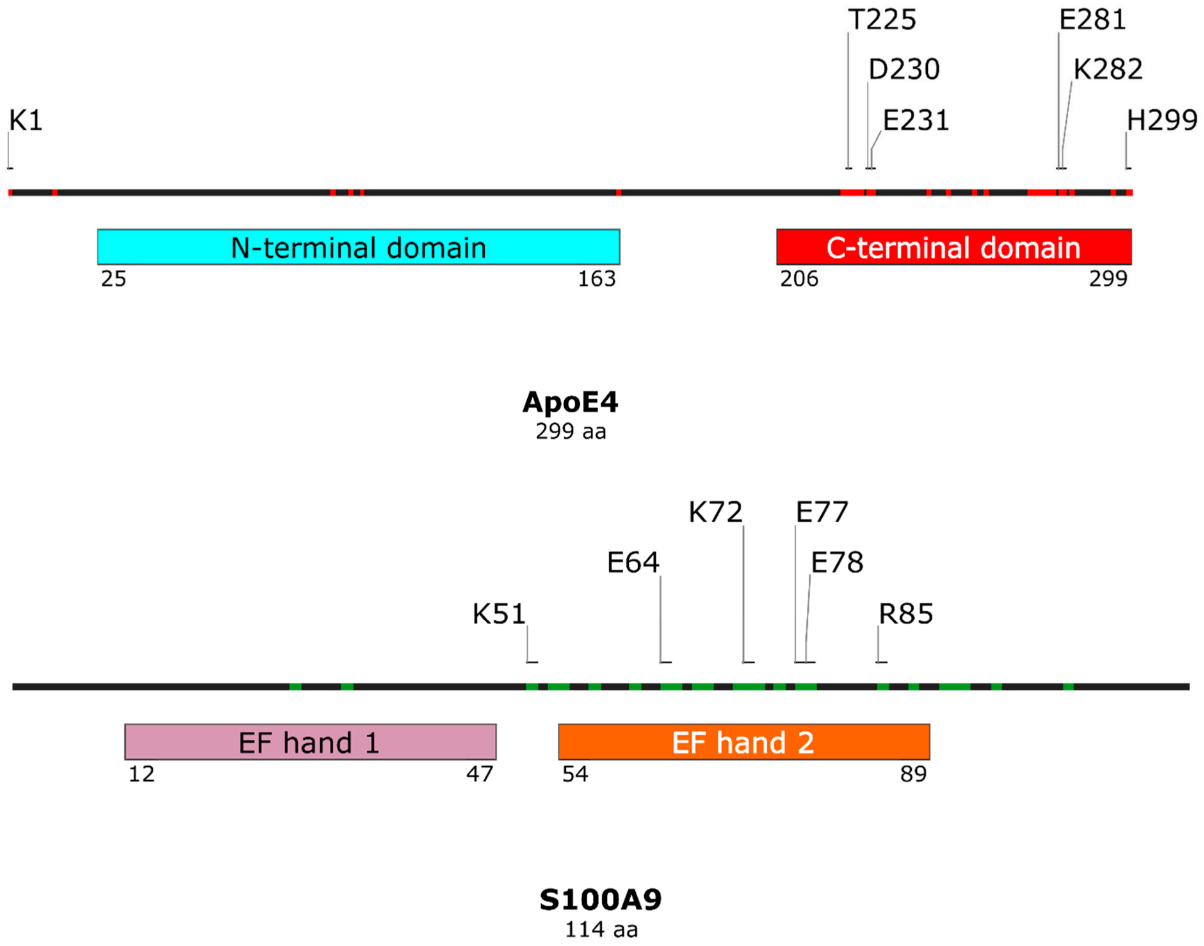

Supplementary Figure S11. Structural pattern diagram demonstrating the amino acid residues involved in S100A9-ApoE complex formation in both proteins. ApoE consists of N-terminal domain, highlighted in cyan, and C-terminal domain, shown in red. S100A9 consists of two EF-hand helix-loop-helix calcium-binding domains, highlighted in brown and orange, respectively, and long unstructured C-terminus. The interacting residues in both proteins determined by MD simulation are indicated individually and located predominantly in the C-terminus of ApoE4 and in the second EF-hand calcium-binding motif of S100A9.

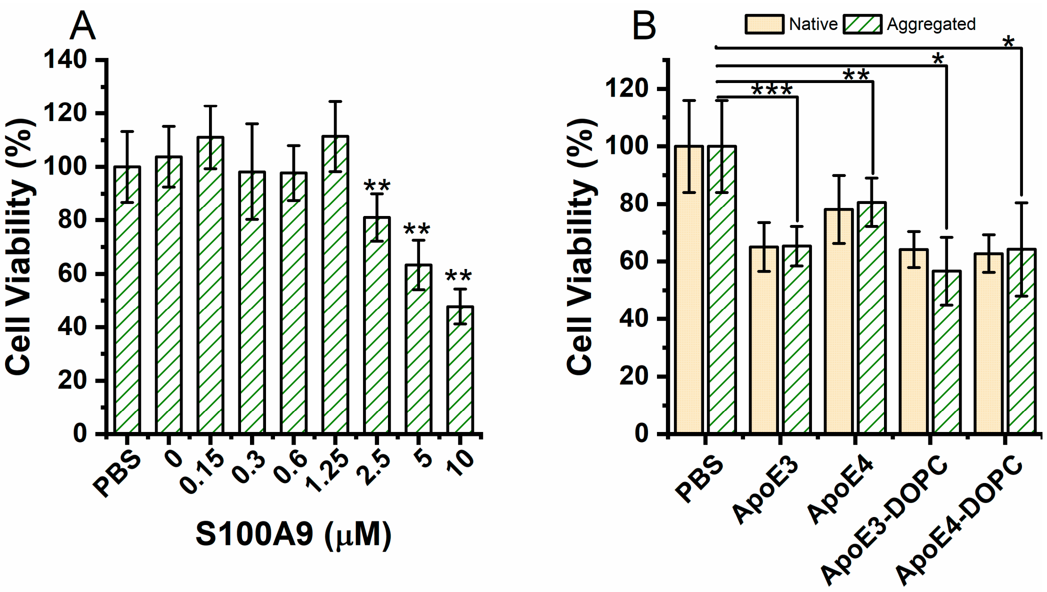

Supplementary Figure S12. Cytotoxicity of individual S100A9 and ApoE on SH-SY5Y neuroblastoma cells. (A) Dose-dependent cytotoxicity of aggregated S100A9. Viability of SH-SY5Y neuroblastoma cells was measured by MTT assay after 24 h co-incubation with increasing concentrations of aggregated S100A9 from 0 to 10 μM. S100A9 was aggregated for 72 h at 42 °C. (B) Viability of SH-SY5Y neuroblastoma cells measured by MTT assay after 24 h co-incubation with lipidated and lipid-free ApoE. Yellow bars correspond to viability of cells treated with freshly SEC purified ApoE protein and green striped bars – to viability of cell treated with ApoE aggregated for 72 h at 42 °C. Different states of ApoE used in the experiment are shown along the *x*-axis. Concentration of ApoE incubated with cells was 500 nM ApoE. *p*-values are indicated as follows: * corresponds to $p<0.05$, ** – $p<0.01$ and *** – $p<0.005$.

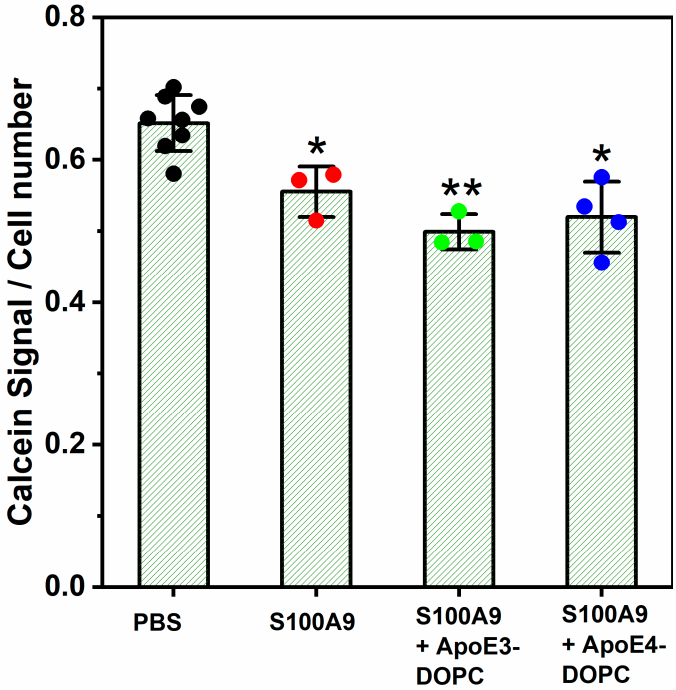

Supplementary Figure S13. Cytotoxicity of S100A9 amyloids incubated in the absence and presence of lipidated ApoE isoforms on SH-SY5Y neuroblastoma cells measured by Calcein-AM assay. Viability of SH-SY5Y neuroblastoma cells was measured after 24 h co-incubation with S100A9 amyloids by monitoring the fluorescence of Calcein-AM and normalizing these values on the Hoechst stained total cell number. The samples used in the experiment are shown below the *x*-axis. 100 µM S100A9 was aggregated in the absence or presence of 5 µM ApoE-DOPC for 72 h at 42 °C. Concentration of S100A9 incubated with cells was 10 µM. *p*-values are indicated as follows: * corresponds to $p<0.05$, ** – $p<0.01$.

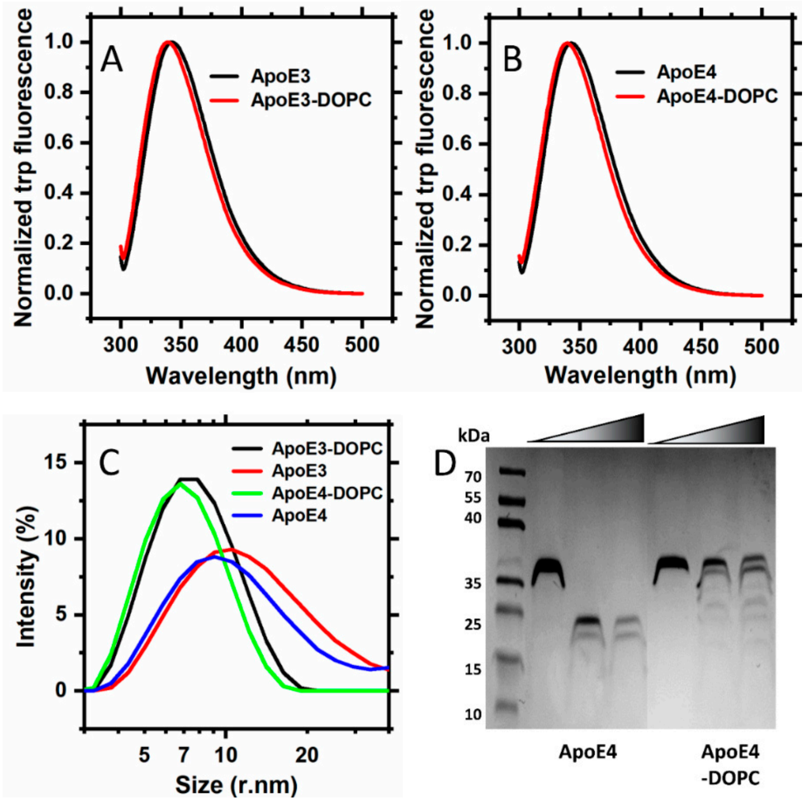

Supplementary Figure S14. Characterization of the fluorescent properties, size and trypsin stability of reconstituted ApoE-DOPC *versus* non-lipidated ApoE. (A) Blue shift of tryptophan fluorescence spectrum of ApoE3 upon lipidation compared to non-lipidated ApoE3 and (B) the same for ApoE4. Both lipidated isoforms of ApoE are characterized by 4 nm blue shift of tryptophan fluorescence (red lines, λ_{max}: 338 nm) versus non-lipidated ApoE isoforms (black lines, λ_{max}: 342 nm). (C) Reduction in hydrodynamic radii of ApoE isoforms upon lipidation determined by dynamic light scattering (DLS). ApoE3-DOPC (black line) and ApoE4-DOPC (green line) are characterized by hydrodynamic radii of 7 nm and 6 nm, respectively, whereas lipid-free ApoE3 (red line) and ApoE4 (blue line) are characterized by hydrodynamic radii of 12 nm and 10 nm, respectively. Probability density function calculated from intensity of scattered light in percent is plotted along *y*-axis. (D) Products of cleavage of ApoE4 and ApoE4-DOPC after 1h incubation with trypsin observed by SDS-PAGE Coomassie blue staining. They are shown as SDS-PAGE bands from left to right: protein molecular weight markers; ApoE4; ApoE4 + Trypsin (1:0.1); ApoE4 + Trypsin (1:0.5); molecular weight markers; ApoE4-DOPC; ApoE4-DOPC + Trypsin (1:0.1) and ApoE4 + Trypsin (1:0.5). Lipid-free ApoE4 was cleaved completely to smaller fragments in 1 h, whereas ApoE-DOPC was more stable and still intact protein was present after 1 h incubation with two concentrations of trypsin. In all the above experiments 5 μM ApoE was used in PBS buffer, 1 mM sodium azide. The experiments are performed at room temperature.

Supplementary Materials and Methods

Calcein-AM staining

Cells were seeded at 80,000 cells/well in black sided 96-well plates (Cell Star, Sigma, USA) coated with 50 µg/mL poly-D-lysine (Sigma-Aldrich, USA) in cell culture medium. After 24 h the cells were washed twice with PBS before addition of 100 µL reduced serum Opti-MEM medium per well (Gibco, Thermo Fisher Scientific, USA). Subsequently, 10 µL of S100A9/ApoE isoform mixture was added to each well and the cells were incubated for further 24 h. Calcein-AM (Invitrogen, USA) was diluted at 5 µg/mL in serum free media and incubated with the cells for 30 min under normal culture conditions. Hoechst 33342 was diluted in serum-free media at a concentration of 5 µg/mL (10X working concentration) and 10 µL dispensed into each well. After 10 min incubation under normal culture conditions, 15 µL of haemoglobin and 200 µL of serum-free media were added to fill each well. Fluorescence images and fluorescent cell counts for Hoechst 33342 (excitation at 405 nm and emission at 450 nm), and Calcein-AM (excitation at 495 nm and emission at 515 nm) were collected for each well using a microplate imager PlateRUNNER HD (Trophos, Roche Group, Switzerland). Calcein-AM results were normalized against Hoechst stained total cell number.

DLS

Lipid-free and lipidated ApoE isoforms were analyzed using DLS. DLS experiments were conducted with a Zetasizer Nano S (Malvern Instruments, Malvern, UK) at 25 °C and using the scattering angle of 173°. 5 µM of purified proteins were used in these experiments. Data were analyzed using an Origin Pro 2020 and represent the averages of 16 acquisitions (10 s per acquisition).

Intrinsic tryptophan fluorescence

The samples of 5 µM of lipid-free and lipidated ApoE isoforms were measured in a 2 mm quartz cuvette using PBS buffer containing 1 mM sodium azide and at room temperature. Intrinsic fluorescence spectra were acquired by using a spectrofluorometer FP 8550 (Jasco Inc, Japan). Excitation at 290 nm and emission between 300 and 500 nm were used with the excitation and emission slits set at 5 nm. The spectra were averaged over three scans recorded at 100 nm/min speed.

Trypsin Cleavage

5 µM of lipidated and lipid-free ApoE were treated with trypsin at the increasing concentrations of 500 nM and 1000 nM for 1 h at room temperature. Then the mixtures were subjected to SDS PAGE as written in main text and stained with Coomassie blue.

Supplementary Table S1. Donor and acceptor pairs, involved in H-bond formation between S100A9 and ApoE4, after 20 µs coarse-grained simulation. Bond criteria were defined as 3 Å length and 20° angle. Ca. 60% occupancy means that in 60% of the frames of the calculated motions in MD the molecular bond was present. Yellow boxes correspond to ApoE residues and green boxes – to S100a9 residues.

H_donor	*H_acceptor*	*occupancy*
THR225-Side-OG1	*GLU64-Side-OE2*	*69,57%*
LYS1-Side-NZ	*GLU78-Side-OE1*	*69,57%*
LYS72-Side-NZ	*GLU231-Side-OE1*	*69,57%*
ARG85-Side-NH1	*HSD299-Side-OT1*	*65,22%*
LYS282-Side-NZ	*GLU78-Side-OE2*	*60,87%*
LYS1-Side-NZ	*GLU77-Side-OE1*	*60,87%*
LYS72-Side-NZ	*ASP230-Side-OD1*	*60,87%*
ARG85-Side-NH2	*GLU281-Side-OE2*	*60,87%*
ARG85-Side-NH2	*HSD299-Side-OT2*	*60,87%*
LYS51-Side-NZ	*GLU281-Side-OE1*	*56,52%*
ARG228-Side-NH1	*GLU77-Side-OE1*	*52,17%*
ASP71-Main-N	*ASP227-Side-OD1*	*52,17%*
ARG85-Side-NH1	*PRO295-Main-O*	*47,83%*
LYS282-Side-NZ	*GLU77-Side-OE2*	*47,83%*
ARG274-Side-NE	*GLU96-Side-OE1*	*47,83%*
GLN258-Side-NE2	*ASP71-Side-OD2*	*43,48%*
ARG226-Side-NH2	*GLU78-Side-OE2*	*43,48%*
ARG228-Side-NH1	*ASP65-Side-OD2*	*43,48%*
ARG228-Main-N	*GLN73-Main-O*	*39,13%*

LYS51-Side-NZ	LEU279-Main-O	39,13%
LYS72-Main-N	ASP227-Side-OD1	34,78%
LYS72-Side-NZ	ASP227-Side-OD2	30,43%
ARG274-Side-NH1	GLU96-Side-OE1	21,74%
ARG251-Side-NE	ASP65-Side-OD2	21,74%
LYS57-Side-NZ	GLU13-Side-OE1	21,74%
ARG226-Side-NE	ASP65-Side-OD1	21,74%
ARG274-Side-NH1	GLU96-Side-OE2	21,74%
GLN246-Side-NE2	HSD103-Main-O	17,39%
ARG228-Side-NH2	ASP65-Side-OD1	17,39%
LYS95-Side-NZ	ASP65-Side-OD1	17,39%
ARG92-Side-NH1	ASP67-Side-OD1	17,39%
TRP88-Side-NE1	TRP276-Main-O	17,39%
ARG251-Side-NH2	HSD61-Side-NE2	17,39%
ASN33-Side-ND2	LEU261-Main-O	17,39%
ARG251-Side-NE	ASP65-Side-OD1	13,04%
ARG251-Side-NH1	HSD61-Side-NE2	13,04%
TRP88-Side-NE1	HSD299-Side-OT2	13,04%
GLN273-Side-NE2	GLU92-Side-OE1	13,04%
LYS57-Side-NZ	GLU13-Side-OE2	13,04%
GLU87-Main-N	HSD28-Side-NE2	13,04%
GLN273-Side-NE2	LYS93-Main-O	13,04%

LYS282-Side-NZ	GLU77-Side-OE1	13,04%
LYS1-Side-NZ	GLU77-Side-OE2	13,04%
LYS1-Main-N	GLU77-Side-OE2	8,70%
GLN284-Side-NE2	GLU78-Side-OE2	8,70%
ARG226-Side-NH1	THR68-Side-OG1	8,70%
ARG226-Side-NH1	GLU64-Side-OE2	8,70%
SER75-Side-OG	ARG228-Main-O	8,70%
LYS72-Side-NZ	ASP227-Side-OD1	8,70%
ARG85-Side-NH2	LYS282-Main-O	8,70%
ARG251-Side-NH2	ASP65-Side-OD1	8,70%
ARG274-Side-NE	GLU96-Side-OE2	8,70%
LYS54-Side-NZ	GLN275-Side-OE1	8,70%
ARG85-Side-NH1	HSD299-Main-C	8,70%
LYS54-Side-NZ	GLN163-Side-OE1	8,70%
ARG224-Side-NH1	ASP71-Side-OD2	8,70%
ARG226-Side-NH2	ASP65-Side-OD1	8,70%
LYS95-Side-NZ	ASP65-Side-OD2	8,70%

ASN53-Side-ND2	*GLY278-Main-O*	*8,70%*
ARG224-Side-NH2	*ASP71-Side-OD2*	*8,70%*
ARG251-Side-NH2	*ASP65-Side-OD2*	*8,70%*
GLN284-Side-NE2	*GLU78-Side-OE1*	*4,35%*
TRP276-Side-NE1	*TRP88-Main-O*	*4,35%*
SER223-Side-OG	*HSD28-Side-NE2*	*4,35%*
LYS51-Side-NZ	*ALA277-Main-O*	*4,35%*
GLN258-Side-NE2	*ASP67-Side-OD2*	*4,35%*
ARG85-Side-NH1	*LYS282-Main-O*	*4,35%*
GLN73-Main-N	*ASP227-Side-OD1*	*4,35%*
TRP276-Side-NE1	*HSD91-Main-O*	*4,35%*
